# Chemosensory Ability and Sensitivity in Health and Disease: Epigenetic Regulation and COVID-19

**DOI:** 10.3390/ijms24044179

**Published:** 2023-02-20

**Authors:** Naina Bhatia-Dey, Antonei B. Csoka, Thomas Heinbockel

**Affiliations:** Department of Anatomy, College of Medicine, Howard University, Washington, DC 20059, USA

**Keywords:** olfaction, gustation, epigenetic and multigenerational inheritance, genome architecture

## Abstract

Throughout the animal kingdom, our two chemical senses, olfaction and gustation, are defined by two primary factors: genomic architecture of the organisms and their living environment. During the past three years of the global COVID-19 pandemic, these two sensory modalities have drawn much attention at the basic science and clinical levels because of the strong association of olfactory and gustatory dysfunction with viral infection. Loss of our sense of smell alone, or together with a loss of taste, has emerged as a reliable indicator of COVID-19 infection. Previously, similar dysfunctions have been detected in a large cohort of patients with chronic conditions. The research focus remains on understanding the persistence of olfactory and gustatory disturbances in the post-infection phase, especially in cases with long-term effect of infection (long COVID). Also, both sensory modalities show consistent age-related decline in studies aimed to understand the pathology of neurodegenerative conditions. Some studies using classical model organisms show an impact on neural structure and behavior in offspring as an outcome of parental olfactory experience. The methylation status of specific odorant receptors, activated in parents, is passed on to the offspring. Furthermore, experimental evidence indicates an inverse correlation of gustatory and olfactory abilities with obesity. Such diverse lines of evidence emerging from basic and clinical research studies indicate a complex interplay of genetic factors, evolutionary forces, and epigenetic alterations. Environmental factors that regulate gustation and olfaction could induce epigenetic modulation. However, in turn, such modulation leads to variable effects depending on genetic makeup and physiological status. Therefore, a layered regulatory hierarchy remains active and is passed on to multiple generations. In the present review, we attempt to understand the experimental evidence that indicates variable regulatory mechanisms through multilayered and cross-reacting pathways. Our analytical approach will add to enhancement of prevailing therapeutic interventions and bring to the forefront the significance of chemosensory modalities for the evaluation and maintenance of long-term health.

## 1. Introduction

**Figure 1:**
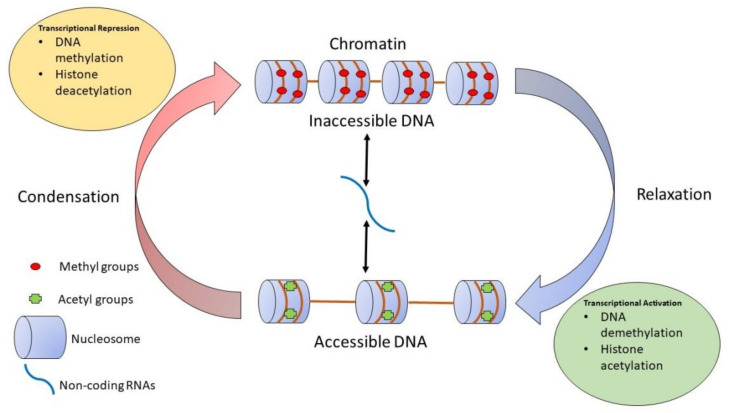
The diagram shows how gene expression is subject to epigenetic regulation. Epigenetic alterations can occur as DNA methylation and/or histone modifications and modify the accessibility of genes to the transcriptional machinery. This takes place either by inducing a relaxed/open or condensed/closed chromatin state. In addition, non-coding RNAs such as miRNAs can regulate the cell phenotype by repressing or enhancing the expression of gene transcripts. These non-coding RNAs can themselves be epigenetically regulated. Reprinted from [3].

All organisms that survive and thrive react to environmental stimuli that impart structural and functional adaptations. Analysis of pathways leading to such experience-induced alterations is a fundamental research topic for clinicians and basic science researchers alike. In an attempt to understand and treat diseases, the field of epigenetics is increasingly receiving attention. Epigenetic changes might contribute to the etiology of diseases such as chemosensory dysfunction. The primary needs of breathing and eating expose organisms to factors which may affect gene regulation and function. At times, such altered regulation becomes a causative force driving multigenerational impact and helps organisms to survive and thrive. In the process, genome function is modulated through epigenetic reprogramming [1]. Epigenetics is a field of study that addresses the relationship between genes, their functional expression and alteration via environmental exposure, and development of pathological features. Epigenetics involves heritable gene expression changes without changes in the DNA sequence itself and affects how the functional trajectory of the same genes is altered as a modulated epigenome alters their expression in cells. Epigenetic modifications are the result of one or more factors, such as age, lifestyle, family history, and disease status. Three major epigenetic modifications have been described: DNA methylation, histone modifications, and non-coding RNA (ncRNA)-associated gene silencing. Epigenetic modifications can occur during an organism’s lifetime and can also be multigenerational, being transmitted to the next generation [2]. As illustrated in Figure 1, and as we have described previously [3], epigenetic regulation involves genome accessibility to the transcriptional machinery; an apparatus that is vulnerable to spatial and temporal deregulation as an outcome of interactions with pathogens and pollutants from the living environment [3]. As shown in Figure 1, methylation at the DNA level, along with histone deacetylation, represses transcription of condensed chromatin because transcription factors cannot access DNA binding sites occupied by methyl groups. Both molecular processes, programmed DNA demethylation and histone acetylation, are amenable to diverse factors that participate in deregulatory pathways. Altered gene expression is detectable at the cellular and organ level resulting from the impact of specific environmental factor/s. This links environmental exposure to diverse diseases through specific alteration of physiological pathways [4]. In this review, we provide background information on gustatory and olfactory dysfunction, followed by a discussion of COVID-19 in relation to our chemical senses. We will then address SARS-CoV-2 infection pathways and immune response at the cellular level. The next section is dedicated to experience, environment, and epigenetics, while another section follows to review olfaction and inheritance.

## 2. Olfactory and Gustatory Dysfunction

While the genetic component primarily contributes to the quality of both olfactory and gustatory abilities in an organism, these chemosensory modalities have plasticity that changes with age, nutritional status, pathological conditions, traumatic experience, diet, as well as additional yet undefined factors [5,6,7,8,9,10]. For instance, one study, involving a community-dwelling aging population in the U.S., identified a relatively higher proportion, approximately 14.8%, of gustatory dysfunction compared to olfactory dysfunction which was approximately 2.7% [11]. The interaction between smell and taste impairment has been well documented. Odors can stimulate olfactory sensory neurons in the olfactory epithelium via two different pathways. In one such pathway known as orthonasal stimulation, odorant molecules are inhaled through the nose and directly reach the olfactory epithelium. In the other pathway known as retronasal stimulation, odorant molecules emanate from food during eating and drinking and reach the olfactory epithelium through the back of the throat during exhalation [12]. Orthonasal and retronasal olfactory perception are functionally synced to the respiratory cycle [13,14,15]. Orthonasal olfactory stimulation takes place during inhalation, whereas retronasal olfactory stimulation occurs during exhalation. Gustatory sensation is accomplished in the mouth through stimulation of taste receptor cells in taste buds housed in papillae on the tongue and soft palate of the oral cavity. Human gustatory sensation has been evaluated typically along with olfactory sensation. Since food also releases odorant molecules, it activates retronasal olfactory stimulation and contributes to the flavor or aroma of food. Gustation and olfaction are known to interact such that modification of one sense leads to changes in the other sense and vice versa. As a result, research studies that exclusively address gustatory dysfunction are absent from the literature. However, the gustatory sense remained unperturbed with transient olfactory impairment of short duration, whereas decreased gustation was clearly associated with long-lasting impaired olfaction [16]. While activated gustatory receptors relay our sense of taste, gustatory cues are augmented by additional sensations through retronasal olfactory stimulation which adds to mutual chemosensory interactions between smell and taste [17]. In healthy adults, an increasing body mass index (BMI) has been associated with a decline in olfactory and gustatory sensitivity [18]. However, BMI impact also indicates an age-dependent variation. In adolescents with higher BMI, there is greater odor sensitivity than in those adolescents that are in early puberty and have a normal BMI. During late puberty, a gradual decline in odor sensitivity becomes apparent [19]. Indeed, olfactory perception has been related to food neophobia in adolescents [20]. Moreover, clinical studies indicate that smell and taste disorders comprise a significant part of adverse drug reactions to pharmacological treatment [21,22]. Research in olfactory genetics indicates diversity and individual differences in olfactory receptor biology. It is further indicative of a differential impact of genetic effects in aging individuals as well as individual resilience to environmental effects accumulated over the lifespan [23]. These findings are suggestive of an elaborate network of pathways that could possibly influence these two sensory modalities individually or in a combinatorial manner. 

## 3. COVID-19 and Our Chemical Senses

Recent clinical analyses of coronavirus disease 2019 (COVID-19) patients have indicated disturbances in the chemical senses as a manifestation of the pandemic [24,25,26,27,28,29,30]. Either impairment or loss of smell and taste have become clear symptoms of COVID-19 [31,32,33,34,35,36,37]. An earlier study using a small but significant number of paucisymptomatic Italian patients revealed ageusia and anosmia as the first and only symptoms of COVID-19 [27]. Most patients recover from COVID-19-induced chemosensory dysfunction, but their recovery rate is variable. In more than 7% of patients, chemosensory disturbances affecting gustation and olfaction persist even after 60 days of first onset of symptoms [27]. Another study revealed that approximately 25% of patients show lingering chemosensory dysfunction after 28 days of first infection [36]. A potentially progressive pattern appears to emerge in the variability of the degree of taste and smell dysfunction [38]; increasing variability might be indicative of multiple regulatory pathways affecting these two sensory modalities. We will try to address such progressive variability in recovery of chemosensory function based on the available data, providing mechanistic insight on pathways and progression of infection. 

As most COVID-19 patients experience loss of smell and taste without nasal congestion and discharge, their anosmia is thought to be the outcome of localized sensorineural damage involving sustentacular (supporting) cells in the olfactory epithelium [37,39,40]. Severe acute respiratory syndrome coronavirus-2 (SARS-CoV-2), the virus responsible for COVID-19, has been found in these support cells. The observation is based on the expression of viral entry proteins angiotensin-converting enzyme 2 (ACE2) and transmembrane protease serine 2 (TMPRSS2) [41,42,43]. For infection to occur, the virus uses ACE2 protein as the cell surface receptor for binding to its spike protein [44]. The next step is facilitated by the proteolytic action of host proteases such as TMPRSS2 [43]. Thus, SARS-CoV-2 uses ACE2 receptors for endocytic host cell entry and TMPRSS2 for spike glycoprotein (S protein) priming and activation [43], which results in direct damage of the olfactory epithelium [39]. The immune system responds to the SARS-CoV-2 virus with hyperactivity and an excessive inflammatory reaction [45]. During COVID-19 infection, proinflammatory cytokine levels are strongly elevated, resulting in a cytokine storm which gives rise to the infiltration of activated immune cells that further interfere with the olfactory system [45,46]. A study involving objective screening of olfactory and gustatory dysfunction in COVID-19 patients indicates that damage to the olfactory system is the primary contributor to symptoms of taste loss rather than viral damage to taste receptors [47] However, the idea has been challenged in subsequent studies. Since the ACE2 receptor, the facilitator of SARS-CoV-2 invasion, is expressed in both neuroepithelium and taste buds [48,49], this is a plausible mechanism of direct impairment of taste-bud receptors by SARS-CoV-2 [47]. However, another study using functional testing of various gustatory modalities in COVID-19 patients failed to detect bona fide hypogeusia [50]. While many COVID-19 patients in this study presented with a loss of taste, this was interpreted as caused by impaired retronasal olfaction. Olfactory dysfunction impacts patients’ lives and psychology [51,52]. Except for olfactory training which involves 15-min interval sniffing of four different odorants (essential oils) twice a day, until now, no effective treatment for COVID-19-related olfactory dysfunction has been found [53,54]. Olfactory training has been used in conjunction with testing the ability for odor discrimination, odor identification, and odor threshold. Age did not affect the results of olfactory training. The odor thresholds improved for several but not all essential oils, whereas the results regarding improvement of discrimination and identification of odors were not clear, suggesting that sensing smell is separate from recognizing identity of odor or requires more training [54]. Post-viral infection-related olfactory loss has already been documented as post-infection outcome for some time [55]. However, its prevalence without rhinorrhea and nasal congestion suggests that this specific virus may follow a distinct pathway to affect its targets rather than the pathways utilized by common cold viruses [40]. A number of explanations have been proposed regarding mechanistic aspects of COVID-19-related anosmia and its possible impact/infection in the brain [56]. Based on the neuro-invasive potential of other corona viruses [57], the cause of COVID-19-related anosmia could be the direct infection, injury, and death of neuronal cells [37,58]. Microvascular injury in the olfactory bulb of COVID-19 patients [59], viral infection of ACE2-expressing vascular pericytes, immune-mediated vascular damage to the olfactory bulb and olfactory mucosa and resulting inflammation can also possibly lead to anosmia [37]. Altered function of olfactory sensory neurons due to infection, damage, and death of supporting cells, microvillar cells, vascular pericytes along with inflammation-mediated mechanisms leading to airflow obstruction are all likely contributors to ansomia [37,60]. Single-cell RNA-sequencing analysis reveals significantly higher expression of ACE2 receptors in epithelial cells of the tongue and, therefore, the buccal mucosa is likely to be susceptible to viral infection as well [49]. The hypothesis that taste disorders of COVID-19 may involve indirect damage of taste receptors through infected epithelial cells and subsequent local inflammation, prevails [37].

A genome-wide association study (GWAS) of COVID-19-related loss of smell and taste of self-reported participants revealed a significant locus in the vicinity of the UGT2A1 and UGT2A2 genes [61]. These two genes belong to the uridine diphosphate glycosyltransferase family and encode enzymes that metabolize lipophilic substrates. Both genes are expressed in the olfactory epithelium. The enzymes eliminate odorants in the nasal cavity after binding to olfactory receptors, thus clearing odorants and facilitating transient olfactory responses [61,62,63]. While the specific function of UGT2A1/UGT2A2 is not clear in COVID-19-related loss of smell, this study provides a first genetic link between the physiology of infected cells and subsequent functional olfactory impairment. Experimental evidence suggests that damage to the olfactory epithelium and cilia on receptor cells due to viral infection contributes to anosmia without infection of olfactory neurons [37].

## 4. SARS-CoV-2 Infection Pathways and Immune Response at the Cellular Level

SARS-CoV-2 virus infection appears to display distinct characteristic features. A recent study has revealed that anosmia related to SARS-CoV-2 virus infection was associated with viral persistence and continued inflammation as virus replication progressed in the olfactory neuroepithelium of the patients [64]. Shifts in the basal microbiome with acute SARS-CoV-2 infection have also been detected when patients have an increased load of bacterial pathogens; such pathogen abundance leads to rise in viral mRNA load, increased inflammation, and damage to neurons [65].

Both in humans and animal models, SARS-CoV-2 virus or viral antigen have been detected in brain endothelial cells [66], despite scant evidence of direct infection in those cells, as they lack detectable level of ACE2 receptor mRNA or protein [67]. In the central nervous system (CNS), despite widespread evidence of SARS-CoV-2 virus [68], the infection is limited to certain cell types [67]. In some infected cell types, virus replication could be inefficient to abortive where virus-infected cells continuously show altered functional responses without additional follow-up production of virus progeny [69]. This could account for an array of mild to moderate symptoms because outcome of virus infection that alters cell functions depending on virus load, the neuro-virulence and neuro-invasiveness of virus variants and the factors regulating immune functions of the patients and/or infected individuals [70]. Further support for this hypothesis comes from experimental evidence using comparative analysis of cellular effects of SARS-CoV-2 and influenza virus IAV infections, with SARS-CoV-2 inflammation in the olfactory bulb and olfactory epithelium being visible even after more than 30 days of initial infection [71]. At the same time, another independent study has shown that SARS-CoV-2 induced antibody response increased in the first months and could be long-lasting in infected individuals, particularly in those with anosmia/dysgeusia, and might be linked to lingering virus in the olfactory bulb [72]. Indeed, experimental evidence indicates an association of neuroinflammation with qualitative difference of neuro-invasiveness and neuro-virulence specific to different variants of the SARS-CoV-2 virus [70,73]. COVID-19-associated olfactory dysfunction leading to anosmia might reflect constantly evolving dynamic interactions of mis-regulated immune system responses in the olfactory bulb, olfactory mucosa, and virus-induced lesions along the entire olfactory tract [71,74].

## 5. Environment, Experience, and Epigenetics

**Figure 2:**
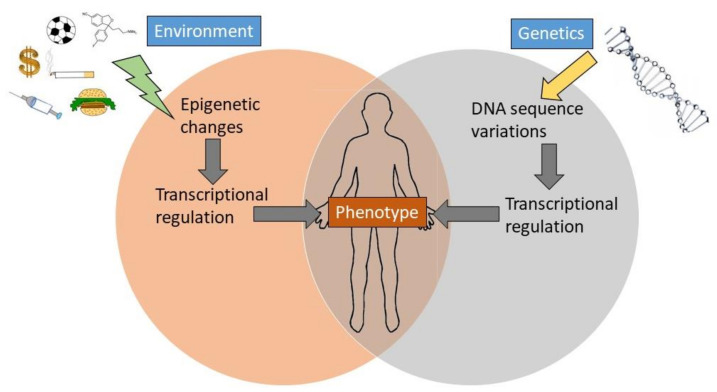
Environmental and genetic influences on the expression of phenotype. Development of polygenic conditions, such as diseases, depend on complex and interacting genetic and environmental pathways. Adapted from [3].

As with other systems, and as we have described previously [2,3], olfactory receptor gene expression is at the interplay of the environment, living conditions and experience. Specifically, the olfactory system is under the influence of learning, fear conditioning, physiological state, social interactions, etc., and, thereby, epigenetics (Figure 2). For example, the presence or absence of odorants affects the sensitivity of the olfactory system as shown for pheromones using a mouse model [75]. Progesterone causes vomeronasal sensory neurons in female mice to be unresponsive to male pheromones during the diestrus stage, but they respond when female mice are in the estrous stage [76,77].

Exposure to odorants stimulates neurogenesis of olfactory sensory neurons despite the fact that increasing age and the environment negatively affect neurogenesis [78]. However, experimental restriction of odorant exposure by closing one nostril in mice leads to selective reduction in specific subtypes of olfactory sensory neurons. This is the result of a change in the rate of generating neurons while their survival rate remains unchanged [78]. The results suggest that an experimental reduction in odorant exposure stimulates neurogenesis of a specific subtype of olfactory neurons, which supports the notion that the mammalian olfactory system can undergo changes in response to the environment.

Olfactory emotional learning has been demonstrated to change the perceptual and cortical representations of previously indiscriminable odor cues in humans [79]. Using aversive and appetitive conditioning in mouse models, it was shown that emotional learning of odor cues alters the primary sensory representation within the nose and brain of adult mice. To illustrate this, transgenic mice were labeled at the M71 odorant receptor, which is specifically activated by the odorant acetophenone. Mice were then behaviorally trained with olfactory-dependent fear conditioning using the odorant acetophenone or conditioned place preference. The mice showed a larger glomerulus (M71-specific) in the olfactory bulb that specifically responded to acetophenone and more odor-specific sensory neurons (M-71-specific) that responded to the odorant compared to mice that were not trained or trained with other odorants [80]. Plasticity and learning in the olfactory system are phenomena that are present in young as well as adult animals, and at the level of olfactory sensory neurons and their projections to the brain. Exposure to odorants can enhance olfactory sensitivity and discrimination and increase the number of new olfactory sensory neurons which supports the notion that smell training can be an effective therapeutic strategy to restore olfactory function after experiencing hyposmia or anosmia as in COVID-19.

There is clear evidence of unique genome-wide methylation profiles for olfactory neuroblastoma tumors [81,82]. Such findings along with evidence of altered cellular function based on transcriptomic data indicate that environmental factors might be modulating the epigenome after viral infection. This phenomenon adds another regulatory layer in the pathology of COVID-19. Nutritional status is a significant factor that influences the internal environment and, thereby, overall cellular function. Tissue-specific altered cellular activity is likely to perturb the microbiome. Since SARS-CoV-2 infections cause shifts in the basal microbiome affecting the severity of COVID-19 symptoms [65] as well as severity and variability of anosmia and dysgeusia, nutrient availability and intake could affect autophagy by modulating neurogenesis [83].

In the animal world, the sense of olfaction is directly correlated with successful foraging, social interactions, fear conditioning and protective cues. In humans, environmental odors impact the activation of the autonomic nervous system, influence perception of stress, and impact avoidance behavior and fear [84,85,86]. Akin to their canine relatives, humans also possess odor tracking abilities [87], and their individual olfactory perception is suggestive of significant non-olfactory genetic information pertaining to age-dependent immunological function [88]. It is an established fact that developmental stage, age and gender are significant modifiers of human olfaction [89,90,91], as is genetic makeup [92,93]. For instance, people with isolated congenital anosmia show somewhat enhanced social anxiety with increased risk of development of depressive symptoms [92,93,94] and the sense of olfaction has been established as a marker of depression using female subjects [94]. Environmental modifiers include odorant exposure, infections, as well as social factors [68,95,96]. Indeed, specific adaptations of olfactory sensory neurons based on their exposure to odorants are evident at the cellular and molecular level in experiments with a mouse model [95]. At the histopathological level, severe neuronal loss in the choroid plexus and other brain regions has been detected in a toddler that succumbed to SARS-CoV-2 infection [68]. In adult patients that had faced childhood maltreatment and were suffering with symptoms of depression, decline in olfactory function was also accompanied by decline in volume of olfactory bulb [96].

## 6. Olfaction and Inheritance

Using the specificity of olfactory molecules, researchers have examined the underlying mechanism of the observed phenomenon of inheritance of parental traumatic exposure in rodent models [97]. Exposure of a known activator of the mouse odorant receptor *Olfr151* in the parental generation of FO and subsequent analysis of behavioral sensitivity to the same odorant in subsequent F1 and F2 generations, revealed enhanced neuroanatomical representation of the Olfr151 pathway along with evidence of CpG hypomethylation (CpG: 5′-C-phosphate-G-3′) in both parental and F1 gametes [97]. This study demonstrated a trans-generational effect on a fear-conditioned olfactory system that was mediated by epigenetic regulation through the sperm of the sire. Similar changes in olfactory sensitivity can be obtained by rewarding appetitive conditioning [80]. Additionally, repeated exposure to an odorant can lead to the induction of enhanced olfactory sensitivity [98]. This was shown when study participants were exposed to several odorants for which they had average baseline sensitivity. Significant changes in olfactory acuity followed in repeated test exposures to the odorants. The increased sensitivity was gender specific such that it was observed only among females of reproductive age, indicating greater olfactory sensitivities in females, possibly related to reproductive behavior [98].

In rodents, odorant receptor proteins localized in olfactory sensory neurons of the olfactory epithelium lining the nasal cavity, are the first molecules exposed to inhaled odorants [99]. In contrast to developing olfactory sensory neurons that transiently express multiple olfactory receptor genes, mature olfactory sensory neurons strictly follow a one-neuron–one-olfactory receptor expression pattern [100,101]. Advancing the experiment with traumatic experience further, a comparative analysis of induced odor fear along with foot shock and then extinguishing the fear with odor exposure only, revealed that F1 offspring were fear extinguished showing lack of behavioral sensitivity towards the exposed odors [102]. At the cellular level, fear-extinguished F1 offspring lacked enhanced representation of M71 or MOR23 odorant receptors in the olfactory system that was evident in fear-trained F1 offspring. Mice were trained with odorants that specifically activated these receptors [102]. Analysis of the methylation level at promoters for genes encoding *Olfr151* and *Olfr160* receptors revealed decreased methylation in F0-trained gametes (F0-trained male mice were trained to associate acetophenone or hydroxymethylpentylcyclohexenecarboxaldehyde (Lyral) presentations with mild foot shocks) compared to F0-exposed gametes (odor presentations were not accompanied by foot shocks) [102]. Similar hypomethylation was evident with another odorant that affected the *Olfr16* promoter. Promoters in F0-trained and F0-extinguished gametes (mice exposed to 30 presentations of the conditioning odor but without foot shocks) were less methylated in comparison to F0-exposed gametes [102]. These findings indicate that application of interventional and perhaps therapeutic strategies in the parental generation is effective either in prevention and/or in reversal of transmission of intergenerational influence of negative experience, harmful exposure, and/or stress.

**Figure 3:**
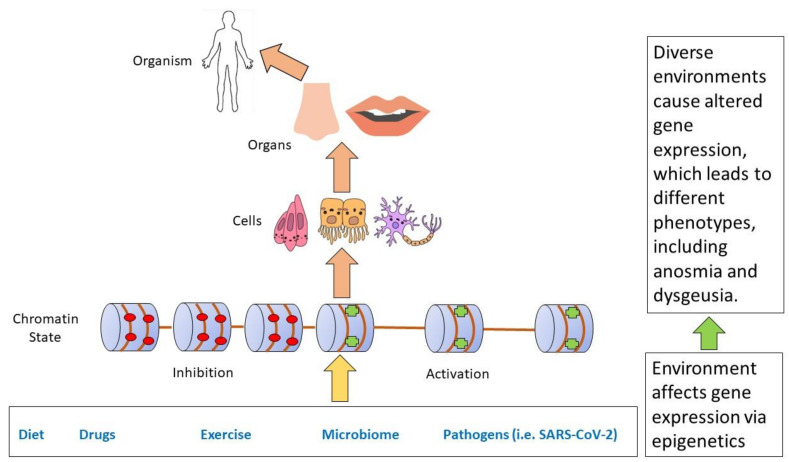
Gene-environment interactions during development and disease occur at different levels. Environmental factors can lead to either inhibition or enhancement of gene expression by incorporating these factors through epigenetic processes such as chromatin remodeling. While the environmental effects manifest themselves initially at the cellular level, they are then incorporated at all levels from cells to the whole organism. Disease etiology occurs in the same hierarchical sequence. In this particular case, SARS-CoV-2 alters epigenetic state contributing to anosmia and dysgeusia. Adapted from [3].

Identifying and mapping transcriptomic clusters to different glomeruli by single-cell RNA-sequencing in developing *Drosophila* olfactory sensory neurons indicates that these neurons use diverse regulatory mechanisms to coordiante their physiology and connectivity through a wide range of expressed transcription factors [103]. Similar regulatory mechanisms are likely to exist in rodents and humans, making the olfactory system susceptible to epigenetic influences. As we have described previously, these could be such diverse influences as traumatic experience, various environmental factors, and in this particular case pathogenic, specifically SARS-CoV-2 infection (Figure 3) [3].

Adding to these observations of extended gustatory and olfactory dysfunction in a group of COVID-19 patients, it is plausible that viral infection alters the designated methylation at promoters of certain sets of affected olfactory receptor genes, thereby leading to prolonged anosmia-hyposmia along with dysgeusia. A supporting clue to this hypothesis is the recommendation of oral and/or nasal steroid treatment to reset the normal olfactory receptor function as well as to test those patients for auto-antibodies with specificity to olfactory recptors [104]. Moreover, a computation analysis of reconstructed chromatin ensembles of olfactory receptors obtained from COVID-19 patients and control samples indicates structural modifications in COVID-19 patients on different levels of chromatin organization [105]. Specifically, there is evidence of pathology-associated changes on already established and known regulatory elements [105]. Indeed, a role of the epigenome in COVID-19-related anosmia has begun to emerge.

## 7. Discussion

Our review of the published literature and analysis of the interactions of environmental factors with the chemical senses indicate a higher degree of vulnerability for these senses as a function of diverse effects of living conditions, including altered histopathology due to bacterial and viral infections. In day-to-day living, exposure to odorants imparts a significant impact on the autonomic nervous system with a decline in olfactory processing, perception of stress, and its resulting physiological outcomes [85,86,106,107]. Indeed, odorants come under the category of strong environmental stimuli, specifically in humans; and the sense of olfaction has a crucial impact on overall behavior, including feeding [108].

Some progress has been made in unraveling the underlying mechanisms of viral infection-associated chemical sense disorders at the cellular and molecular levels. Histopathological analysis reveals that persistent anosmia and olfactory dysfunctions that continue several weeks after SARS-CoV-2 virus infection are accompanied by an altered ratio of olfactory sensory neurons and support cells, and aberrant gene expression. This alteration appears to reflect continued inflammatory signaling [109]. Analysis of gene expression profiles of olfactory epithelium from autopsies of COVID-19 patients shows that SARS-CoV-2 virus infection correlates with significant downregulation of olfactory receptor transcripts as well as the transcripts for olfactory receptor signaling genes [74]. Effects of SARS-CoV-2 virus infection on nuclear architecture are non-cell autonomous and significantly affect odor perception [74].

As discussed above, anosmia and dysgeusia associated with COVID-19 correlate with a diverse range of symptoms that also vary based on physiological, genetic and environmental factors [110,111,112]. A consistent finding is the presence of widespread inflammation and altered expression of inflammation-related signaling molecules [19,64,74]. In fact, throughout the biological kingdom, inflammation has been known to provide crucial protection from adverse effects of environmental factors to maintain the functional and structural integrity at the cellular level [113]. The possibility of some degree of adult neurogenesis in the olfactory bulb and olfactory neuroepithelium of COVID-19 patients could be a mechanism to compensate for the cellular loss caused by virus-induced autophagy. A similar autophagic hypothesis has been postulated to explain age-related decline of olfactory processing in mice [114].

In terms of therapeutic interventions, phytochemicals have emerged as effective agents that could facilitate the recovery of COVID-19-induced anosmia and ageusia [115]. An additional therapeutic approach could involve the regulation of signaling pathways associated with Neuropilin-1 and semaphorins, which are the key molecules participating in gustatory and olfactory axon guidance [116]. Future research aimed at understanding their impact on recovery of post-infection chemical senses would possibly evaluate epigenetic alterations that might account for resistance to infection, and reduced impact on olfactory and gustatory dysfunction and/or loss.

## 8. Concluding Comments

Among the major effects of SARS-CoV-2 virus infection is the change in both olfactory and gustatory function/sensibility. Multiple factors regulate post-infection alterations in the chemical senses, and include physiological, environmental as well as genetic and epigenetic factors.

Increased abundance of bacterial pathogens in nasal epithelium and evidence of a specific shift in the nasal microbiome of severe COVID-19 patients indicate an additional environmental factor contributing to COVID-19 symptoms. Disruption of the epigenome through altered methylation and acetylation could impart prolonged, or at times, permanent disruption of the chemical senses. In addition, preliminary evidence from computational analysis of reorganized chromatin further suggests active participation of the epigenome.
